# The Role of Vitamin E in Protecting against Oxidative Stress, Inflammation, and the Neurotoxic Effects of Acute Paracetamol in Pregnant Female Rats

**DOI:** 10.3390/toxics11040368

**Published:** 2023-04-12

**Authors:** Alaa M. Hammad, Baraa Shawaqfeh, Suhair Hikmat, Tariq Al-Qirim, Lama Hamadneh, Sameer Al-Kouz, Mariam M. Awad, Frank S. Hall

**Affiliations:** 1 Department of Pharmacy, College of Pharmacy, Al-Zaytoonah University of Jordan, Amman 11733, Jordan; 2 Department of Basic Medical Sciences, Faculty of Medicine, Al-Balqa Applied University, Al-Salt 19117, Jordan; 3 Department of Pharmacology and Experimental Therapeutics, College of Pharmacy and Pharmaceutical Sciences, University of Toledo, Toledo, OH 43606, USA; frank.hall@utoledo.edu

**Keywords:** APAP, *Cyp1a2*, *Cyp2d6*, *Nat2*, ALT, AST

## Abstract

Paracetamol (acetaminophen, APAP) is the most common non-prescription analgesic drug used during pregnancy. The aim of this study was to investigate the effect of vitamin E on acute APAP toxicity in pregnant rats. Toxicity in the liver, kidney, and brain (hippocampus, cerebellum, and olfactory bulb) was examined. Twenty pregnant female Wistar rats at gestational day 18 were used. Pregnant rats were divided into four groups: Control, APAP, E + APAP, and APAP + E. The Control group was treated with 0.5 mL p.o. corn oil. The APAP group received 3000 mg/kg p.o. APAP. The E + APAP group received 300 mg/kg p.o. vitamin E one hour before 3000 mg/kg APAP. The APAP + E group received 3000 mg/kg paracetamol one hour before 300 mg/kg p.o. vitamin E. Twenty-four hours after the last treatment administration, rats were euthanized and blood, brain, liver, and kidney samples were collected. Alanine aminotransferase (ALT), aspartate aminotransferase (AST), blood urea nitrogen (BUN), creatinine levels, uric acid (UA), and superoxide dismutase (SOD) levels, as well as the relative mRNA expression of *Cyp1a4*, *Cyp2d6*, and *Nat2*, were determined. Acute APAP treatment upregulated ALT, AST, BUN, and creatinine levels. APAP treatment downregulated UA and SOD levels. APAP treatment upregulated the relative mRNA expression of *Cyp1a4* and *Cyp2d6*, but downregulated *Nat2* expression. Vitamin E treatment, either before or after APAP administration, attenuated the toxic effects of APAP. In conclusion, the results showed that an acute toxic APAP dose in late pregnancy can cause oxidative stress and dysregulation in Cyp isoform expression, and that vitamin E treatment attenuates these effects.

## 1. Introduction

Acetaminophen, also known as paracetamol (N-acetyl-para-aminophenol; APAP), is an over-the-counter painkiller and antipyretic that is safe when used at the recommended levels (no more than 4 gm/day) for adults, including pregnant women, as well as children (no more than 75 mg/kg/day). Self-medication with APAP is commonly used for fever and pains associated with common upper respiratory infections, e.g., rhinoviruses, influenza viruses, and many others, including COVID-19, for which it has been widely used during the pandemic in Jordan [1,2]. APAP overdoses, both accidental and purposeful, are common and frequently result in hepatotoxicity [3,4,5]. Nephrotoxicity and neurotoxicity are less common in APAP overdoses, but can occur independently or concurrently with hepatotoxicity [6,7]. Importantly, a previous study showed that APAP is the most common over the counter (OTC) drug associated with toxicity during pregnancy [8,9], so it is especially important to examine APAP toxicity under these conditions. Thus, in this study, we used pregnant female rats to investigate the effect of an acute toxic dose on toxicity in different organs during pregnancy.

APAP directly alters hormone-dependent processes in vivo, in vitro, and ex vivo, thereby affecting neural and reproductive development in both sexes [10]. In rodents, fetal APAP exposure has been experimentally demonstrated to produce male urogenital tract reproductive problems, including abnormalities in testicular function, sperm, and sexual behavior [11,12,13]. According to several studies, female ovarian development is also disrupted by APAP, resulting in fewer oocytes, early ovarian insufficiency, and decreased fertility [14,15]. It has been shown that exposure to fetal APAP alters neurotransmission in the brain, resulting in changes to cognition, behavior, and movement [16]. The results of these investigations have demonstrated that the impact of APAP is influenced by the timing of exposure in relation to particular developmental processes, as well as length of the exposure. For further information, see [10].

APAP is metabolized in the liver through either phase I or phase II metabolism [17]. Phase II metabolism is the major pathway, starting with UDP-glucuronosyl transferases (UGT) which conjugate glucuronic acid to APAP to form a glucuronic acid conjugate (a nontoxic metabolite) [18]. The sulfuric acid conjugate is the second nontoxic metabolite that is formed by the action of sulfotransferase (SULT). The phase I pathway involves CYP450 enzymes, including *Cyp1a2* and *Cyp2d6*, that de-acetylate APAP to form N-acetyl-p-benzoquinone imine (NAPQI) (a toxic metabolite) that is normally detoxified by glutathione (GSH) to form mercaptate conjugates (nontoxic metabolites) at non-toxic APAP doses [19,20]. Furthermore, since APAP shares structural similarities with endogenous acetylated products, it decreases N-acetyltransferase 2 (NAT2) function, which has been shown both in vitro and in vivo [21]. Acute APAP toxicity overproduction of NAPQI depletes hepatic GSH reserves, resulting in the accumulation of reactive oxygen species (ROS), oxidative stress, mitochondrial dysfunction, DNA fragmentation, and ultimately, hepatocyte apoptosis or necrosis [22,23,24].

Many studies have also reported increased levels of creatinine and blood urea nitrogen (BUN) after APAP toxicity [25,26,27]. These outcomes are potential indicators of nephrotoxicity. Paracetamol-induced nephrotoxicity also results in metabolism of APAP to form NAPQI and then p-aminophenol (PAP) in the kidney by de-acetylation. PAP converts to a reactive quinone by the action of prostaglandin endoperoxide synthase [26,28], thereby resulting in nephrotoxicity.

Although not a common outcome reported for APAP overdose, neurotoxicity might also result. Several studies have detected the presence of CYP450 enzymes within the brain that would most likely metabolize APAP to the toxic metabolite NAPQI. These enzymes are mainly found in the olfactory bulbs, olfactory cortex, hippocampus, cerebellum, and brain stem [29,30,31]. APAP metabolism in the brain to the toxic metabolite NAPQI leads to an increase in *Cyp2e1* levels and neuronal cell death [32]. Nevertheless, the currently available studies on the effects of acute APAP toxicity on the brain are sparse and none of them have measured CYP450 expression in many brain regions. Other studies have shown that during APAP toxicity, ROS generation and GSH depletion occurs [33].

The primary purpose of this work was to assess the potential effects of acute APAP toxicity in pregnant rats. Several measures of toxicity were examined, including the production of ROS in the liver, kidney, and brain, as well as the expression of CYP450 isozymes in the liver, kidney, and brain. This study also investigated the therapeutic effects of vitamin E against APAP-induced toxicity in the liver, kidney, and brain in pregnant rats. Vitamin E (α-tocopherol) is a lipid-soluble small molecule antioxidant that belongs to the class of tocopherol antioxidants. Vitamin E is the most potent form that can neutralize reactive nitrogen species (RNS), including nitric oxide (NO), nitrogen dioxide (NO_2_), and peroxy-nitrite. Vitamin E has many sources, including common food oils produced from corn, peanuts, and soybeans [34]. Consequently, we investigated the pre- and post-treatment protective effects of vitamin E on an acute toxic APAP dose in pregnant rats.

## 2. Materials and Methods

### 2.1. Animals

This study was conducted on 20 female Wistar rats weighing 200 ± 10 g at the start of the study. Wistar rats were obtained from the Al-Zaytoonah University of Jordan animal program. The animals were kept in a 12/12 hr light/dark cycle at room temperature (25 ± 2 °C) and humidity (45 ± 5%), with free access to food and water. The animal protocol (protocol #18/06/2018–2019) for this work was approved by the Animal Care and Use Committee of Al-Zaytoonah University of Jordan and all work was conducted in accordance with the Helsinki guidelines for animal research [35] and all applicable Jordanian governmental rules and guidelines.

### 2.2. Drugs

APAP powder (UC448) (≥97%) and vitamin E (T3251) (89.0–102.0%) were purchased from Sigma Aldrich, Burghausen, Germany.

### 2.3. Experimental Procedure

Twenty female Wistar rats, average weight 200 ± 10 g, were housed in cages alone after mating. Mating consisted of placing two virgin females and one male together prior to the end of the daily light cycle. The following morning, each female was examined for the presence of a vaginal plug. Females were separated after vaginal plug detection and housed alone. Pregnant females were placed in separate cages and kept on a 12:12 light/dark cycle. The day after mating was taken as day zero of pregnancy (gestational day zero, GD0). Abdominal enlargement of female rats was observed on day 16 after mating, which was taken as a positive indicator of pregnancy. GD19 was chosen to take samples for this experiment because pregnancy is visually apparent at this stage and GD19 is representative of late-stage pregnancy in rats, equivalent to the second trimester in humans. On GD17, all animals were fasted overnight. On GD18, rats were divided into 4 groups (5 rats per group): Group I (Control) received 0.5 mL p.o. corn oil (vehicle), Group II (APAP) received a single dose of 3000 mg/kg p.o. APAP dissolved in corn oil, Group III (E + APAP) received a single dose of 3000 mg/kg p.o. APAP dissolved in corn oil one hour after 300 mg/kg p.o. vitamin E dissolved in corn oil, and Group IV (APAP + E) received a single dose of 3000 mg/kg p.o. APAP dissolved in corn oil one hour before 300 mg/kg p.o. vitamin E dissolved in corn oil. All groups were euthanized twenty-four hours after the treatments (GD19). Blood was collected in heparinized tubes and stored at −20 °C, and the brain, kidney, and liver were collected and snap frozen for further analysis. High doses of APAP were used to ensure toxicity in liver, kidney, and brain, as shown by previous studies [36,37,38]. Additionally, high doses of vitamin E were used to measure pre- and post-treatment protective effects as reported previously [39,40,41].

### 2.4. Blood Biochemistry: Hepatotoxicity

Blood samples (around 1 mL) were taken from the aorta of each rat, collected in heparinized tubes, and were centrifuged at 4000 rpm for 10 min. Plasma was obtained from the samples using a calibrated micropipette and stored at −20 °C until subsequent analysis for the levels of ALT, AST, serum creatinine (SCr), and BUN.

#### 2.4.1. Alanine Aminotransferase (ALT)

ALT analyses were conducted using the manual procedure of the Biolabo ALT analysis kit (REF#80027, Biolabo S.A.S., Les Hautes Rives, France). A reduction in absorbance in the samples is due to the conversion of NADH to NAD^+^ and is proportional to ALT activity in the specimen. The absorbance was measured at 340 nm after 1, 2, and 3 min. The absorbance at each time point was subtracted from the previous measurement (e.g., the value for time-point 2 minus the value for time-point 1, and the value for time-point 3 minus the value for time-point 2). The absolute values of these two measurements were averaged and converted to a measure of absorbance rates (ΔAbs/min). ALT levels were calculated using the following equation by comparing the absorbance in the sample to the absorbance in a calibration standard:

$$\mathrm{ALT}\;\mathrm{levels}\;(\mathrm{IU}/L)=\frac{Sample\;Absorbance(\Delta abs/min)}{Standard\;Absorbance(\Delta abs/min)}\times \mathrm{standard}\;\mathrm{concentration}$$

#### 2.4.2. Aspartate Aminotransferase (AST)

AST analyses were conducted using the manual procedure of the Biolabo AST analysis kit (REF#80025, Biolabo S.A.S., Les Hautes Rives, France). Decreases in absorbance in the sample reflect the conversion of NADH to NAD^+^ and are proportional to AST activity in the sample. The absorbance was measured at 340 nm after 1, 2, and 3 min. The absorbance for each time point was subtracted from the previous measurement (e.g., the value for time-point 2 minus the value for time-point 1, and the value for time-point 3 minus the value for time-point 2). The absolute values of these two measurements were averaged and converted to a measure of absorbance rates (ΔAbs/min). The AST levels were calculated using the following equation by comparing the absorbance in the sample to the absorbance in a calibration standard:

$$\mathrm{AST}\;\mathrm{levels}\;(\mathrm{IU}/L)=\frac{Sample\;Absorbance(\Delta abs/min)}{Standard\;Absorbance(\Delta abs/min)}\times \mathrm{standard}\;\mathrm{concentration}$$

### 2.5. Blood Biochemistry: Nephrotoxicity

#### 2.5.1. Serum Creatinine (SCr)

Serum creatinine analyses were conducted using the manual procedure of the Bioloabo creatinine kinetic method kit (REF#80107, Biolabo S.A.S., Les Hautes Rives, France). Creatinine levels were measured by an analysis of the absorbance reduction. Creatinine reacts with picrate to form a colored complex in alkaline solution. Creatinine levels were measured at 490 nm at 30 (A_1_) and 150 (A_2_) seconds. Serum creatinine levels were calculated using the following equation:

$$\mathrm{Serum}\;\mathrm{Creatine}\;\mathrm{levels}\;(\mathrm{mg}/\mathrm{dL})=\frac{Sample\;Absorbance\left(A_{2}-A_{1}\right)}{Standard\;Absorbance\left(A_{2}-A_{1}\right)}\times \mathrm{standard}\;\mathrm{concentration}$$

#### 2.5.2. Blood Urea Nitrogen (BUN)

BUN levels were analyzed using the manual procedure of the Biolabo urea kit (REF#80221, Biolabo S.A.S., Les Hautes Rives, France). BUN analyses depend on the hydrolysis of urea to ammonium ions and carbon dioxide by the action of urease. The ammonium ions form chloride and salicylate complexes that are blue-green in color, which correlate with the urea concentration in the specimen. The absorbance is determined at 600 nm. BUN levels were calculated using the following equation:

$$\mathrm{Serum}\;\mathrm{BUN}\;\mathrm{levels}\;(\mathrm{mg}/\mathrm{dL})=\frac{Sample\;Absorbance}{Standard\;Absorbance}\times \mathrm{standard}\;\mathrm{concentration}$$

### 2.6. Brain Tissue Harvesting

Twenty-four hours after treatments, rats were euthanized by diethyl ether (#673811, Sigma Aldrich, Burghausen, Germany) overdose and decapitated using a guillotine. Brains were extracted and frozen in liquid nitrogen prior to storage at −80 °C. Using a cryostat set to −20 °C, tissue samples from the olfactory bulbs, hippocampus, and cerebellum were anatomically identified and dissected according to the rat brain atlas of Paxinos and Watson [42]. Samples were divided for analysis by quantitative polymerase chain reaction (qPCR) for mRNA, uric acid levels, and superoxide dismutase inhibition. All samples were frozen until later analysis.

### 2.7. Tissue Preparation

Kidney, liver, olfactory bulb, hippocampus, and cerebellum tissue samples were isolated and washed with ice-cold isotonic saline (0.9%) and then stored at −80 °C until later processing. Tissue samples were homogenized in 50 mM phosphate buffer (PH = 7.4) using an electronic homogenizer polytron PT1200 Kinematica AG^®^, Eschbach, Germany) to prepare 10% (*w*/*v*) homogenates. After centrifugation, supernatants were isolated and aliquots were separated into Eppendorf tubes and stored at −80 °C until assays were conducted for PCR, superoxide dismutase (SOD), and uric acid (UA) measurements.

### 2.8. Antioxidant Status

#### 2.8.1. Uric Acid (UA)

UA levels in liver, kidney, and brain samples were detected using the Biosystem uric acid kit (REF#80351, Biosystem^®^, Barcelona, Spain). The kit forms a colored complex (red color) that can be measured at 520 nm. Uric acid levels were calculated using the following equation:

$$\mathrm{Uric}\;\mathrm{acid}\;\mathrm{levels}\;(\mathrm{mg}/L)=\frac{Sample\;Absorbance}{Standard\;Absorbance}\times \mathrm{standard}\;\mathrm{concentration}$$

#### 2.8.2. Superoxide Dismutase (SOD)

SOD activities in liver, kidney, and brain samples were detected using the Abcam superoxide dismutase activity kit (ab65354; Abcam, Cambridge, UK). Analyses of SOD activity depend on a rection with the tetrazolium salt WST-1 that produces a water-soluble formazan dye upon reduction with a superoxide anion. The activity is determined as the difference between the activity of the sample and control (blank) samples. Blanks 1, 2, and 3, as well as the samples, were prepared according to Table 1.

The absorbance was measured at 450 nm. SOD activity, expressed as % inhibition, was calculated using the following equation:

$$\%\;\mathrm{SOD}\;\mathrm{Inhibition}=\frac{\left[\left\{\mathrm{Absorbance}\left(\mathrm{Blank}\;1\right)-\mathrm{Absorbance}\left(\mathrm{Blank}\;3\right)\right\}-\left\{\mathrm{Absorbance}\left(\mathrm{sample}\right)-\mathrm{Absorbance}\left(\mathrm{Blank}\;2\right)\right\}\right]}{\left[\left\{\mathrm{Absorbance}\left(\mathrm{Blank}\;1\right)-\mathrm{Absorbance}\left(\mathrm{Blank}\;3\right)\right\}\right]}\times 100$$

### 2.9. Real-Time, Quantitative PCR

Total RNA was isolated from the tissue samples using the Thermo Scientific, GeneJet RNA Purification Kit (cat# K0732) according to the manufacturer’s protocol. Ten micrograms of total RNA from each sample was used for cDNA synthesis. Reverse transcription (RT) was conducted using a Thermo Scientific verso cDNA synthesis kit (cat#AB1453B) according to the manufacturer’s protocol. RNA and cDNA concentrations were measured using a Nanodrop Quawell DNA/Protein Analyzer (Thermo Fisher Scientific, Sunnyvale, CA, USA). cDNA samples were diluted to a total concentration of 100 ng/μL, and 1 μL of each diluted cDNA sample was used for a quantitative real-time polymerase chain reaction (qRT-PCR) according to the manufacturer’s protocol. qRT-PCR was performed using a reaction mixture of TAKARA Cyber Green as a fluorescent dye (TP Green Premix Ex Taq II), a 1/20 volume of cDNA as a template, and the appropriate primers for the genes of interest. A threshold cycle number (CT) for each sample was obtained from an iCycler thermal cycler (Bio-Rad Laboratories, München, Germany) and used to compare the relative amount of target mRNA in experimental groups with those of controls, using the 2^−ΔΔCT^ method [43]. Each sample was run in triplicate. To calculate ΔCT, the mean CT value for the control gene (GAPDH) was subtracted from the mean CT value of the gene of interest. The ΔCT values for group C were then averaged and subtracted from ΔCT for each measurement from the experimental groups to obtain ΔΔCT. The relative fold changes from the control were then expressed by calculating 2^−ΔΔCT^ for each sample.

### 2.10. Statistical Analysis

Graphpad Prism 9.0 software was used for statistical analyses. The results were expressed as means ± standard deviation (SD). Values between groups were compared using a one-way ANOVA followed by Tukey post hoc tests to determine statistical significance between individual means.

## 3. Results

### 3.1. Blood Biochemistry: Hepatotoxicity

The hepatic function was assessed by measuring the levels of ALT and AST.

#### 3.1.1. Alanine Aminotransferase (ALT)

**Figure 1:**
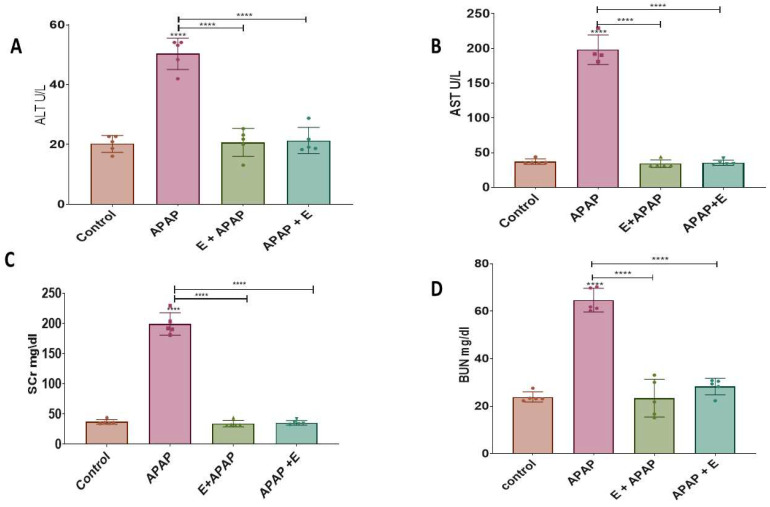
Blood biochemistry showing hepatotoxicity and nephrotoxicity after a single acute APAP (3000 mg/kg) administration and vitamin E (300 mg/kg) treatment (means ± SEM): (**A**) ALT levels, (**B**) AST levels, (**C**) SCr levels, and (**D**) BUN levels (****: *p* < 0.0001, *n* = 5 for each group). Individual data points are shown in the figure.

APAP increased the ALT levels compared to the control group. Either pre-treatment or post-treatment administration of vitamin E attenuated this effect of APAP (Figure 1A). This effect was confirmed by a one-way ANOVA showing a significant main effect of treatment (F (3, 16) = 57.37, *p* < 0.0001). Tukey multiple comparisons showed a significant elevation in ALT after treatment with 3000 mg/kg p.o. APAP in comparison to the control group, while pre-treatment and post-treatment vitamin E administration prevented this increase. These results indicate that single dose of APAP caused liver toxicity and vitamin E reversed this effect.

#### 3.1.2. Aspartate Aminotransferase (AST)

APAP (3000 mg/kg p.o.) increased AST levels compared to the control group, while pre-treatment or post-treatment administration with vitamin E attenuated this effect (Figure 1B). This effect was confirmed by a one-way ANOVA showing a significant main effect of treatment (F (3, 15) = 261.8, *p* < 0.0001). Tukey multiple comparisons showed a significant elevation in AST after treatment with 3000 mg/kg p.o. compared to the control group, while pre-treatment or post-treatment vitamin E administration prevented this increase. These results indicate that APAP administration caused lever toxicity and vitamin E reversed this effect.

### 3.2. Blood Biochemistry: Nephrotoxicity

The kidney function was assessed by measuring the levels of creatinine and BUN.

#### 3.2.1. Serum Creatinine (SCr)

APAP (3000 mg/kg) increased SCr levels compared to the control group, while pre-treatment or post-treatment administration of vitamin E attenuated this effect (Figure 1C). This effect was confirmed by a one-way ANOVA showing a significant main effect of treatment (F (3, 16) = 331.4, *p* < 0.0001). Tukey multiple comparisons showed a significant elevation in SCr after treatment with 3000 mg/kg p.o. in comparison to the control group, while pre-treatment or post-treatment vitamin E administration prevented this increase. These results suggest that APAP caused nephrotoxicity and vitamin E reversed this effect.

#### 3.2.2. Blood Urea Nitrogen (BUN)

APAP (3000 mg/kg) increased BUN levels compared to the control group, while pre-treatment or post-treatment administration of vitamin E attenuated this effect (Figure 1D). This effect was confirmed by a one-way ANOVA showing a significant main effect of treatment (F (3, 16) = 75.45, *p* < 0.0001). Tukey multiple comparisons showed a significant elevation in BUN after treatment with 3000 mg/kg p.o. APAP in comparison to the control group, while pre-treatment or post-treatment administration of vitamin E prevented this increase. These results suggest that APAP caused nephrotoxicity and vitamin E reversed this effect.

### 3.3. Antioxidant Status

The results for the determination of uric acid (UA) and superoxide dismutase (SOD) activities in liver, kidney, and brain samples are shown below.

#### 3.3.1. Uric Acid (UA)

**Figure 2:**
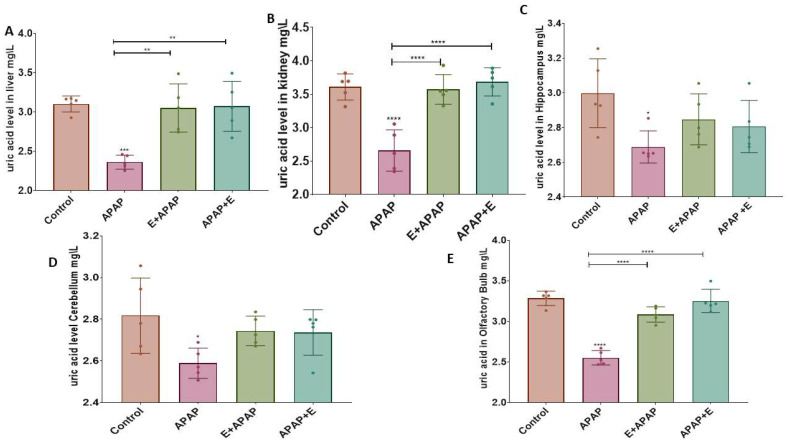
UA levels in different organs and brain regions after a single acute APAP (3000 mg/kg) administration and vitamin E (300 mg/kg) treatment (means ± SEM): (**A**) liver, (**B**) kidney, (**C**) hippocampus, (**D**) cerebellum, and (**E**) olfactory bulbs (*: *p* < 0.05, **: *p* < 0.01, ***: *p* < 0.001, ****: *p* < 0.0001, *n* = 5 for each group). Individual data points are shown in the figure.

APAP (3000 mg/kg p.o.) decreased UA concentrations in the liver and kidney compared to the control group, while pre-treatment or post-treatment vitamin E administration attenuated this effect (Figure 2A and Figure 2B, respectively). These effects were confirmed by a one-way ANOVA, which showed the main effect of treatment in the liver (F (3, 16) = 11.95, *p* = 0.0002) and the kidney (F (3, 16) = 20.65, *p* < 0.0001). Tukey multiple comparison tests showed a significant decrease in UA after treatment with 3000 mg/kg p.o. APAP in comparison to the control group, while pre-treatment and post-treatment vitamin E administration prevented this increase. In addition, APAP decreased UA concentrations in the hippocampus and cerebellum compared to the control group (Figure 2C and Figure 2D, respectively). This effect was confirmed by a one-way ANOVA, which showed significant main effects of treatment in the hippocampus (F (3, 16) = 3.555, *p* = 0.0383) and the cerebellum (F (3, 16) = 3.347, *p* = 0.0456). Tukey multiple comparisons showed a significant decrease in UA after treatment with 3000 mg/kg p.o. APAP in comparison to the control group. These decreases were very modest and comparisons to the vitamin E-treated groups did not demonstrate a significant difference from APAP treatment alone. However, in the olfactory bulbs APAP decreased the UA concentration compared to the control group. This effect was more obvious than the changes seen in the other brain regions and pre-treatment or post-treatment administration of vitamin E significantly attenuated this effect (Figure 2E). This effect was confirmed by a one-way ANOVA, which showed the significant main effect of treatment (F (3, 16) = 50.49, *p* < 0.0001). Tukey multiple comparisons showed a significant decrease in UA after treatment with 3000 mg/kg p.o. APAP in comparison to the control group, while vitamin E administration prevented this decrease.

#### 3.3.2. Superoxide Dismutase (SOD)

**Figure 3:**
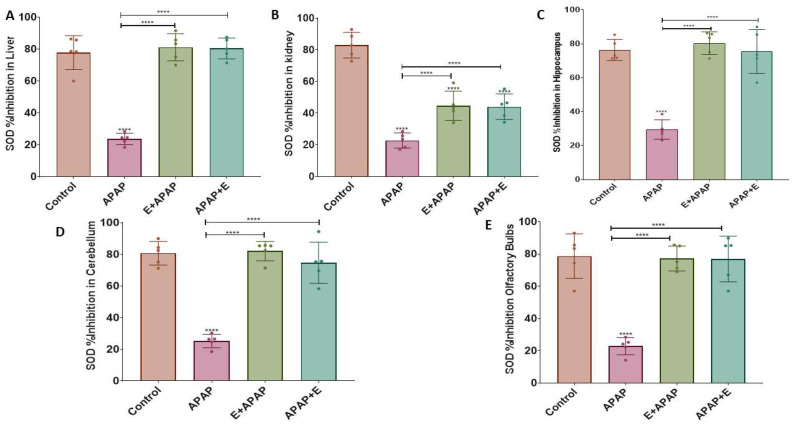
SOD inhibition (%) in different organs and brain regions after a single acute APAP (3000 mg/kg) administration and vitamin E (300 mg/kg) treatment (mean ± SEM): (**A**) liver, (**B**) kidney, (**C**) hippocampus, (**D**) cerebellum, and (**E**) olfactory bulbs (****: *p* < 0.0001, *n* = 5 for each group). Individual data points are shown in the figure.

APAP (3000 mg/kg p.o.) decreased the SOD concentration in the liver, kidneys, hippocampus, cerebellum, and olfactory bulb compared to the control group, while pre-treatment or post-treatment vitamin E administration attenuated this effect (Figure 3A, Figure 3B, Figure 3C, Figure 3D and Figure 3E, respectively). These effects were confirmed by a one-way ANOVA, which showed the significant main effects of treatment in the liver (F (3, 16) = 65.34, *p* < 0.0001), the kidneys (F (3, 16) = 52.65, *p* < 0.0001), the hippocampus (F (3, 16) = 41.10, *p* < 0.0001), the cerebellum (F (3, 16) = 52.11, *p* < 0.0001), and the olfactory bulb (F (3, 16) = 31.28, *p* < 0.0001). Tukey multiple comparisons showed a significant decrease in SOD concentration after treatment with 3000 mg/kg p.o. APAP in comparison to the control group, while vitamin E administration eliminated or reduced this decrease in all organs/brain regions that were compared.

### 3.4. Real-Time Quantitative PCR Results:

The results for determination of the relative expressions of *Cyp1a4*, *Cyp2d6*, and *Nat2* mRNA in liver, kidney, and brain samples are shown below.

#### 3.4.1. *Cyp1a4*

**Figure 4:**
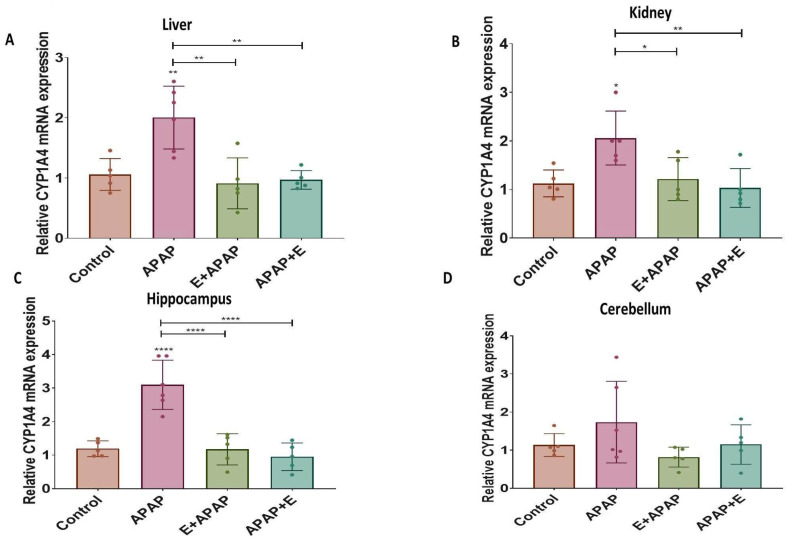
Relative *Cyp1a4* mRNA expression in different organs and brain regions after single acute APAP (3000 mg/kg) administration and vitamin E (300 mg/kg) treatment (mean ± SEM): (**A**) liver, (**B**) kidney, (**C**) hippocampus, and (**D**) cerebellum (*: *p* < 0.05, **: *p* < 0.01, ****: *p* < 0.0001, *n* = 5 for each group). Individual data points are shown in the figure.

APAP (3000 mg/kg p.o.) increased the relative *Cyp1a4* expression in the liver, kidneys, and hippocampus compared to the control group, while pre-treatment or post-treatment vitamin E attenuated this effect (Figure 4A,B and Figure 3C, respectively). These effects were confirmed by a one-way ANOVA, showing the main effects of treatment in the liver (F (3, 17) = 10.54, *p* = 0.0004), the kidney (F (3, 16) = 6.063, *p* = 0.0059), and the hippocampus (F (3, 17) = 21.86, *p* < 0.0001). Tukey multiple comparisons found significant increases in the relative *Cyp1a4* expression after treatment with 3000 mg/kg p.o. APAP in comparison to the control group, while pre-treatment and post-treatment vitamin E administration prevented these increases. However, the relative *Cyp1a4* expression in the cerebellum was not significantly different (Figure 4D), although it did show a similar pattern.

#### 3.4.2. *Cyp2d6*

**Figure 5:**
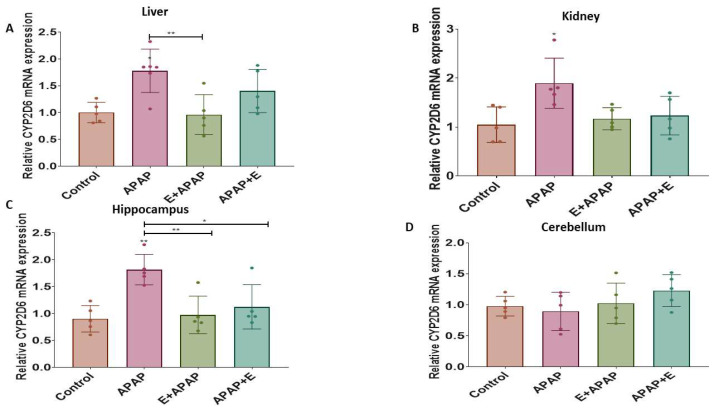
Relative *Cyp2d6* mRNA expression in different organs and brain regions after single acute APAP (3000 mg/kg) administration and vitamin E (300 mg/kg) treatment (mean ± SEM): (**A**) liver, (**B**) kidney, (**C**) hippocampus, and (**D**) cerebellum (*: *p* < 0.05, **: *p* < 0.01, *n* = 5 for each group). Individual data points are shown in the figure.

APAP (3000 mg/kg p.o.) increased the relative *Cyp2d6* expression in the liver, kidneys, and hippocampus compared to the control group. Vitamin E pre-treatment attenuated this effect in the liver and the hippocampus, while vitamin E post-treatment attenuated this effect in the hippocampus (Figure 5A, Figure 5B and Figure 5C, respectively). These effects were confirmed by a one-way ANOVA, which found the main effects of treatment in the liver (F (3, 17) = 6.41, *p* = 0.0042), the kidney (F (3, 16) = 4.84, *p*= 0.0139), and the hippocampus (F (3, 16) = 8.11, *p* < 0.0016). Tukey multiple comparison tests found significant increases in the relative *Cyp2d6* expression after treatment with 3000 mg/kg p.o. APAP in comparison to the control group. Pre-treatment vitamin E administration prevented this increase in the liver, while pre-treatment and post-treatment vitamin E administration prevented this increase in the hippocampus. However, the relative *Cyp2d6* expression in the cerebellum was not significantly affected by the treatments (Figure 5D).

#### 3.4.3. Nat2

**Figure 6:**
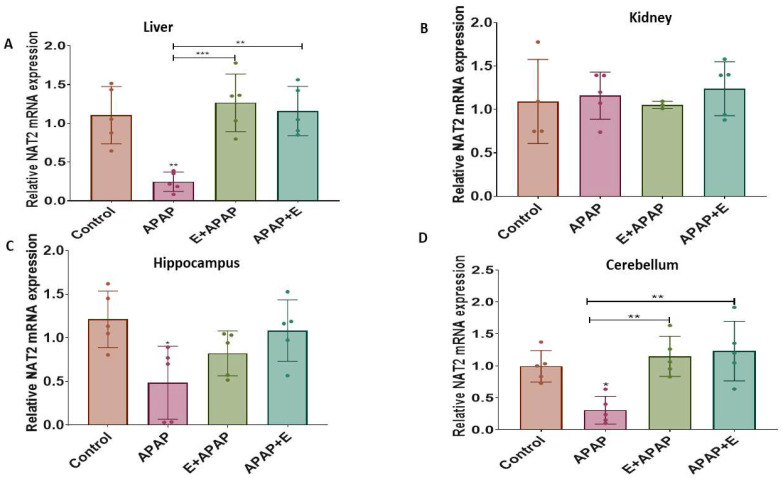
Relative *Nat2* mRNA expression in different organs and brain regions after a single acute APAP (3000 mg/kg) administration and vitamin E (300 mg/kg) treatment (mean ± SEM): (**A**) liver, (**B**) kidney, (**C**) hippocampus, and (**D**) cerebellum (*: *p* < 0.05, **: *p* < 0.01, ***: *p* < 0.001, *n* = 5 for each group). Individual data points are shown in the figure.

APAP (3000 mg/kg p.o.) decreased the relative *Nat2* expression in the liver, hippocampus, and cerebellum compared to the control group, while pre-treatment or post-treatment vitamin E attenuated this effect in the liver and the cerebellum (Figure 6A, Figure 6C and Figure 6D, respectively). These effects were confirmed by a one-way ANOVA, showing the main effects of treatment in the liver (F (3, 17) = 11.29, *p* = 0.0003), the hippocampus (F (3, 16) = 4.38, *p*= 0.0198), and the cerebellum (F (3, 16) = 8.36, *p* = 0.0014). Tukey multiple comparison tests found significant decreases in the relative *Nat2* expression after treatment with 3000 mg/kg p.o APAP in comparison to control group, while pre-treatment and post-treatment vitamin E administration in the liver and the cerebellum normalized the *Nat2* levels. A similar pattern was observed in the hippocampus, but the post hoc comparisons did not find significant differences. The relative *Nat2* expression in the kidney was not significantly different between treatment groups (Figure 6B).

## 4. Discussion

This study examined acute APAP toxicity in the liver, kidney, and brain. These studies focused on pregnant rats, since APAP is the most commonly used analgesic during pregnancy. Moreover, this study investigated the potential benefits of prophylactic and therapeutic vitamin E treatment for preventing APAP-induced hepatic, renal, and neural toxicity. Acute APAP poisoning is frequently linked to hepatic and, to a lesser extent, renal inflammation, which occurs primarily as a result of cellular oxidative stress and pro-inflammatory immunological responses [44]. Our results showed that the levels of ALT and AST were significantly increased in the APAP-treated group in comparison to the control group, indicating that APAP induced hepatic injury. These results are consistent with a previous study that found a significant elevation in ALT and AST levels after administration of 750 mg/kg APAP to male rats [45]. Another study also showed that 3000 mg/kg p.o. APAP increased the levels of ALT and AST in male rats [46]. This elevation was explained by impaired transport function in hepatocytes, resulting in leakage of the plasma membrane, thereby increasing enzyme levels in the serum [45]. In order to investigate the in vivo protective effects of vitamin E, ALT and AST levels were determined in the present study and a significant improvement in both biochemical parameters were observed after either pre-treatment or post-treatment with vitamin E. Similarly, a previous study showed a restoration in ALT and AST levels after treatment with 50 mg/kg vitamin E for 6 weeks before treatment with 3000 mg/kg p.o. APAP [47].

In this study, the levels of creatinine and BUN were significantly increased in rats treated with APAP compared to the control group, indicative of kidney injury. These results agree with observations in previous studies that showed that levels of serum creatinine and BUN were elevated 24 h after administration of 1000 mg/kg i.p. APAP to male rats [48,49]. Moreover, similar to the observations here, in pregnant female rats, elevated creatinine and BUN levels were reduced by vitamin E administration either pre-treatment or post-treatment. Similarly, a previous study in mice also showed that pretreatment with 30 mg/kg p.o. vitamin E before 900 mg/kg i.p. APAP restored the creatinine and BUN levels to normal [50].

APAP produced other evidence of hepatic, renal, and neural (hippocampus, cerebellum, and olfactory bulb) toxicity as shown by a significant decline in UA and SOD levels, suggesting the presence of oxidative stress. Similar results were reported in a previous study, which examined the level of oxidative biomarkers such as catalase and SOD in rat kidneys following administration of 750 mg/kg/day APAP for 7 days [51]. Furthermore, another study found reduced hepatic antioxidant markers after administration of 2 g/kg p.o. APAP to mice in terms of catalase and SOD functions [52]. Other studies have demonstrated significant reductions in catalase and SOD in the entire brain of male mice after administration of 600 mg/kg i.p. APAP [53,54].

To investigate the protective effects of vitamin E treatment, given either before or after APAP in pregnant female rats, the present study examined uric acid and SOD levels in the liver, kidney, hippocampus, cerebellum, and olfactory bulb. Vitamin E attenuated the harmful effects of acute APAP administration on hepatic, renal, and neural (hippocampus and cerebellum) toxicity, as shown by the measures of oxidative stress. Moreover, significant improvements in uric acid and SOD levels were shown in the liver, kidney, hippocampus, cerebellum, and olfactory bulb after vitamin E treatment. This is consistent with one study showing that pretreatment with 30 mg/kg p.o. vitamin E before 900 mg/kg i.p APAP in BALB/c mice of both sexes restored the antioxidant status to normal levels [50].

APAP causes acute toxicity primarily through the metabolism of APAP to NAPQI by CYP enzymes. APAP is metabolized by the CYP isoforms *Cyp2e1*, *Cyp1a4*, and *Cyp2d6* [55,56,57]. An additional pathway for APAP-induced acute toxicity is through deacetylation of APAP by N-deacetylase enzymes to PAP, which is a toxic metabolite [58]. Following the production of PAP, it can be reconverted into APAP by NAT2 [59]. In the present study, the relative mRNA expressions of *Cyp1a4*, *Cyp2d6*, and *Nat2* were measured. A single dose of 3000 mg/kg p.o. APAP to pregnant female rats upregulated the relative mRNA expressions of *Cyp1a4* and *Cyp2d6*, while the expression of *Nat2* was reduced in all organs except in the kidney. *Nat2* RNA expression is low in the kidney compared to the liver and even lower in female kidneys compared to male kidneys, which might make detecting reductions in *Nat2* expression in the kidney more difficult [60]. More studies are warranted to evaluate this finding. These changes in enzyme expression are consistent with previous studies that reported that the relative mRNA expressions of some CYP isoforms are upregulated after a single oral dose of APAP [44,61]. The present study showed that pre-treatment or post-treatment vitamin E administration attenuated CYP mRNA dysregulation caused by APAP. This result is in line with several reports on the protective effects of vitamin E through effects that normalize CYP isoform function and/or expression [62,63,64].

The present study examined pregnant female subjects because APAP is commonly used by pregnant women, and APAP overdose occurs at a fairly high rate, perhaps because users perceive it to be relatively safe drug. The present study expanded upon our knowledge of the mechanisms underlying APAP overdoses, both in terms of the role of alterations in the expression of metabolizing enzymes and the effects on measures of neurotoxicity. The present study did not examine non-pregnant female rats or male rats, so it is difficult to say whether pregnancy or sex influences the toxicity of APAP or the protective effects of vitamin E. Comparisons across previous studies do not provide answers because the conditions (dose and treatment regimens and other factors) differ substantially between studies. Thus, answers to these questions will require further, specific study. It should also be noted that the effects of these drugs on in utero toxicity were not explored here but given the findings here on toxicity in pregnant dams, in utero toxicity might be of some concern, and, at the very least, worthy of study as well.

There are a few additional limitations to this study. In future studies, a positive control group treated with N-acetyl cysteine (NAC), the standard treatment for acetaminophen toxicity [50], should be added to the experimental design in order to compare it to the efficacy of vitamin E. This would be an important comparison for evaluation of vitamin E as an alternative for NAC, which has a high rate of serious anaphylaxis associated with its use [65]. A study by Şener, Şehirli and Ayanoğlu-Dülger [50] showed that NAC was more efficacious than vitamin E, but it remains to be seen whether that was due to the treatment parameters used in that single-dose study. Other parameters of the experiment certainly need additional examination, including the time between exposure and treatment. The present study also showed that pre-treatment with vitamin E reduces APAP toxicity, which should also be investigated, particularly in the context of APAP use in pregnancy. The present studies investigated such pre-treatments under a limited set of circumstances. Future studies should address potential prophylactic uses of vitamin E under more clinically relevant circumstances. Another limitation involves additional investigations of the underlying mechanism, including the effect of APAP on *Cyp2e1*, which is also involved in acetaminophen detoxification. Vitamin E effects on post APAP toxicity survival should also be evaluated. More studies are warranted in order to address these limitations. Additionally, future research should be focused on the effect of acute APAP doses and vitamin E treatment on fetal survival and health.

In summary, this study clearly demonstrated the toxic effects of an acute oral dose of APAP in pregnant female rats. This addresses an important limitation of previous studies that have not addressed overdose in pregnant females. Furthermore, the toxic effects of APAP were shown in the liver and kidney, and for the first time in the brain (cerebellum, hippocampus, and olfactory bulbs). APAP-induced toxic effects were shown for oxidative stress biomarkers, including SOD and UA. Additionally, and for the first time, we showed the effects of APAP on the expression of minor metabolic enzymes, often not examined in toxicity studies, that could contribute to the production of toxic metabolites after APAP administration. Moreover, the many outcomes detailed in the current study reveal the protective effect of vitamin E treatment, either when given prophylactically or after exposure to an acute toxic dose of APAP. These actions of vitamin E protected against hepatotoxicity, nephrotoxicity, and neurotoxicity. The potential for prophylactic use of vitamin E may be especially worthy of future investigation as it would limit the negatives effects associated with APAP use and might potentially prevent the toxic effects before they occur.

## Figures and Tables

**Table 1:** Components of the working solutions in SOD activity procedure.

Component	Sample (µL)	Blank 1 (µL)	Blank 2 (µL)	Blank 3 (µL)
Sample solution	20	0	20	0
ddH_2_O	0	20	0	20
WST working solution	200	200	200	200
Enzyme working solution	20	20	0	0
Dilution Buffer	0	0	20	20

## Data Availability

Not applicable.
